# The Antisense Protein ASP of HIV-1 Enhances Viral Entry in CD4+ T Cells

**DOI:** 10.3390/v17101332

**Published:** 2025-09-30

**Authors:** Myriam Abla Houmey, Isabella Caico, Aurélie Rivault, Lucile Espert, Jean-Michel Mesnard, Fabio Romerio, Nathalie Chazal

**Affiliations:** 1 Institut de Recherche en Infectiologie de Montpellier (IRIM), CNRS, Université de Montpellier, 34090 Montpellier, France; 2 Department of Molecular and Comparative Pathobiology, Johns Hopkins University School of Medicine, Baltimore, MD 21205, USA; 3 Department of Molecular Microbiology and Immunology, Johns Hopkins Bloomberg School of Public Health, Baltimore, MD 21205, USA

**Keywords:** HIV-1, antisense protein, ASP, viral entry

## Abstract

The negative strand of the human immunodeficiency virus-1 (HIV-1) proviral genome contains an antisense open reading frame encoding a protein (ASP) with no known homologs. The presence of immune responses to ASP in people living with HIV-1 (PLWH) demonstrates its expression in vivo. Further, the predicted hydrophobicity of ASP is consistent with its association with the plasma membrane and viral envelope. Despite this body of evidence, the role of ASP in HIV-1 replication remains unknown. In this report, we investigated the hypothesis that the presence of ASP on the viral surface enhances HIV-1 entry into target cells. We generated an ASP-knockout replication-competent HIV-1 molecular clone in the NL4-3 background, which we used to perform cell–cell fusion, viral entry, and viral replication assays. Our results suggest that the presence of ASP on the plasma membrane of infected cells and the envelope of HIV-1 virions enhances viral transmission. Overall, our studies provide first evidence that ASP plays a role in the HIV-1 replication cycle. Further investigation into these observations may lead to the identification of new HIV-1 vulnerabilities that may be the target of novel interventions.

## 1. Introduction

The presence of an antisense open reading frame (ORF) in the negative strand of the human immunodeficiency virus type 1 (HIV-1) proviral genome was first reported more than three decades ago based on conserved start and stop codons in twelve group M viral strains [1]. Subsequent studies provided further evidence of the new gene (termed *asp*) in thousands of viral isolates [2,3,4,5,6]. The ASP ORF overlaps the *env* gene at the surface/transmembrane junction, and it is predicted to encode a protein (ASP) of approximately 189 amino acids from a Tat-independent promoter in the 3′ long terminal repeat [7]. The expression of ASP in vivo has not been directly investigated, but the presence of cellular and humoral immune responses to ASP in peripheral blood of people living with HIV-1 (PLWH) provides compelling indirect proof [8,9,10,11].

Although the function of ASP remains unknown, there is ample evidence to suggest a role in the virus life cycle. The *asp* gene is found in pandemic (group M) HIV-1 strains but not in all other non-pandemic lentiviruses [2], and it originated through nucleotide substitutions that did not significantly impact the sequence of Env, indicating that *asp* is not an accidental byproduct of *env* evolution [3,4]. The presence of the ASP ORF constrains the evolution of *env*, which suggests that conservation of *asp* benefits HIV-1 or it would be rapidly lost to random genetic drift [4]. Further, within group M HIV-1, *asp* is found more frequently in high- compared with low-frequency clades [2], and also in isolates from rapid progressors compared with long-term non-progressors [4]. Finally, ASP co-localizes with gp120 at the plasma membrane, and it has also been detected on the envelope of infectious virions [12]. Recent reports also described patterns of covariation between amino acid substitutions in the variable loop 3 (V3, the major determinant of coreceptor usage) of the envelope glycoprotein gp120 and in ASP that are associated with coreceptor tropism [13,14].

Altogether, these lines of evidence support the notion that ASP may be involved in the process of viral transmission or spread. Here, we explored this hypothesis using fusion and binding assays. Our findings clearly demonstrate that ASP facilitates viral entry in CD4+ T-cell lines and primary CD4+ T lymphocytes.

## 2. Materials and Methods

### 2.1. Cell Culture

Primary CD4+ T lymphocytes were isolated from peripheral blood mononuclear cells and purified by negative selection using the CD4+ T-cell isolation kit (Miltenyi Bio-Tech, Bergisch Gladbach, Germany, 130-096-533). Cells were then activated with anti-CD3 and antiCD28 antibodies (BioXCell, Hanover, NH, USA, BE0001-2 and BE0248, respectively) at 1 µg/mL each in complete RPMI 1640 medium supplemented in 10% fetal calf serum (FCS) and 1% penicillin–streptomycin. After activation, cells were cultured with 100 U/mL of IL-2 (Miltenyi Bio-Tech, Bergisch Gladbach, Germany, 130-097-746) in complete RPMI 1640 medium. All experiments involving primary cells were performed 4 days post-activation.

HuT 78 or HuT 78-GFP cells were cultured and maintained in RPMI 1640 medium supplemented in 10% fetal calf serum (FCS) and 1% penicillin–streptomycin. HEK 293T cells were cultured and maintained in Dulbecco’s modified Eagle’s medium (DMEM) supplemented with antibiotics and 10% FCS. For HEK-Env [15] and HEK-Env-mCherry cells, 2 µM of methotrexate (MTX) was added to the culture to maintain envelope protein expression.

### 2.2. Plasmids and Antibodies

The pNL4-3 HIV-1 proviral DNA was obtained through the NIH HIV Reagent Program, Division of AIDS, NIAID, NIH: human immunodeficiency virus 1 (HIV-1), strain NL4-3 infectious molecular clone (pNL4-3), ARP-114, contributed by Dr. M. Martin. To produce the pNL4.3-ASP Mut12 construct, a NdeI/BamHI fragment containing the *asp* ORF was first cloned into the pGL3 basic vector digested with the same enzymes. Using primers 24-8 (5′GTTGCAACTCACAGTCTGGGGCAT-3′) and 24-7 (5′-AGATGCTGTTGAGCCTCAATAGCC-3′), reverse PCR was used to mutate the cysteine residue at position 12 of the *asp* ORF into a stop codon (TGC into TGA). After insertion of the nucleotide substitution was confirmed by DNA sequencing, the fragment was excised from the pGL3 basic vector and cloned back into pNL4-3 replacing the wildtype segment. As previously described, the coding sequences of ASP, GFP-ASP, and GFP-ASP Mut12 mutant were inserted into the mammalian expression vector pCAGGS [10].

For RRE activity assays, pGag-RRE plasmid is derived by deletion of pol from the pGagPol-RRE (pHR354). This plasmid was then used to generate pGag-RRE Mut12 by site directed mutagenesis [16].

Anti-p24 was purchased from Bio-Rad (ref: 4999-9007). Anti-actin was purchased from Sigma (ref: A5441). Secondary antibodies goat anti-mouse–HRP conjugate (GAM-HRP); goat anti-rabbit–HRP conjugate (GAR-HRP), and donkey anti-goat–HRP conjugate (DAG-HRP) were purchased from Bio-Rad (ref: 1706516, 1706515, and OBT1500P, respectively). The following reagents were obtained through the NIH HIV Reagent Program, Division of AIDS, NIAID, NIH: human immunodeficiency virus 1 (HIV-1) IIIB gp160 monoclonal antibody (Chessie 13-39.1), ARP-1209, contributed by Dr. George K. Lewis; polyclonal anti-human immunodeficiency virus type 1 Vpr protein, residues 1 to 50 (rabbit antiserum, ARP-11836, contributed by Dr. Jeffrey Kopp). Two types of monoclonal antibodies against ASP were used: (i) a monoclonal antibody produced by EUROGENTEC from mice immunized with an HXB2-derived peptide [10] and (ii) a monoclonal antibody against ASP generously provided by Fabio Romerio [12].

### 2.3. Lentiviruses Production and Transduction

To obtain the GFP- or mCherry-expressing lentiviruses, HEK 293T cells were co-transfected with pCMV-VSV-G (Addgene, Watertown, MA, USA, 12259; deposited by Dr. Didier Trono), pPAX2, and pNaldini-GFP [17] or pLVX-mCherry-C1 (Clontech, Mountain View, CA, USA, No. 632561). Lentiviral particles were concentrated by ultracentrifugation on a PBS (Gibco, New York, NY, USA, 14190–094)-25% sucrose (Millipore, Darmstadt, Germany, 84100) cushion and quantified by p24 ELISA (Bio-techne, Minneapolis, MN, USA, DY7360-05) according to the manufacturer’s instructions.

### 2.4. Generation of Stable Cell Lines

HEK-Env cells [16] or HuT 78 were incubated with 1 μg (eq. p24) of lentiviral vectors encoding either mCherry or GFP and either puromycin or Methotrexate (MTX) for HuT78 or HEK-Env cells, respectively. After 48 h of transduction, cells were washed and cultured in complete media containing the appropriate drug for selection of transduced cells.

### 2.5. Viruses

NL4-3 ASP WT, NL4-3 ASP Mut12, NL4-3-BlamVpr-ASP WT, and NL4-3-BlamVpr-ASP Mut12 viruses were produced by transfection of molecular clones with PEImax (Clinisciences, Nanterre, France) into HEK 293T cells. The culture supernatants were collected 48 h post-transfection, filtered through a 0.45 µm membrane (Dutscher ClearLine, Bernolshein, France), and ultra-centrifuged over a 25% sucrose cushion (Millipore) for 2 h at 25,000 rpm. Virus pellets were resuspended in FCS-free DMEM, and viral particles were quantified by p24 ELISA (Bio-techne, DY7360-05) according to the manufacturer’s instructions.

### 2.6. Western Blot

A total of 250 ng (p24 equivalents) of viral stocks were lysed in 1× NuPAGE LDS Sample Buffer (Invitrogen, Waltham, MA, USA, NP0007) supplemented with 1× Bolt Reducing Agent (Invitrogen B0009), heated for 10 min at 95 °C, and loaded on 10% Tris-Tricine gels. Proteins were transferred onto a PVDF membrane at 60 V for 2 h using two transfer buffers: an anode buffer (0.1 M Tris, pH 8.9) and cathode buffer (0.1 M Tris; 0.1 M Tricine; 0.1% SDS; pH 8.25). After blocking for 1 h at room temperature in 0.5% Casein/PBS, membranes were blotted with primary antibody for 1 h at room temperature. Following three washing steps with 0.1%Tween/PBS, membranes were blotted with HRP-conjugated secondary antibody for 1 h at room temperature and washed again as above. ECL (Clarity western ECL Substrate; Bio-Rad, Hercules, CA, USA, 170–5061) was used for detection with a ChemiDoc camera (Bio-Rad). Quantification of protein was performed using the Image lab Software Version 6.1 (Bio-Rad). Data were normalized to p24 expression or actin when mentioned.

### 2.7. Fusion Assay

For fusion assays, HEK-Env-mCherry cells were transfected with 0.5 µg of DNA (pCAGGS or pCAGGS-ASP) using Turbofect (Thermo Scientific, Waltham, MA, USA, 3780621–R0531) according to the manufacturer’s instructions. Twenty-four hours post-transfection, cells were washed with PBS and co-cultured with HuT 78-GFP cells at a 1:1 ratio (0.5 × 10^6^ cells/well total). After co-culture, cells were observed by fluorescence microscopy and formation of syncytia as assessed by the presence of double positive cells was scored by flow cytometry at different time points. As a control, cells were treated for 1 h with 1 µg/mL of the fusion inhibitor, AMD3100 (Sigma, Merck KGaA, Darmstadt, Germany).

### 2.8. Blam-Vpr Assay

Blam-Vpr Assay was performed as previously described [18]. Briefly, viruses containing Blam-Vpr were produced in HEK 293T cells by co-transfection of Blam-Vpr expression vector and molecular clones. Viruses were prepared as described in the “Viruses” Section of the Materials and Methods. A total of 0.5 × 10^6^ HuT 78 or primary CD4+ T cells plated in 24-well plates were infected with 1 µg (eq. p24) of viruses for 3 h at 37 °C. After several washes, cells were loaded with a CCF2-AM loading kit (Invitrogen) at room temperature for 2 h and washed. Another incubation was performed overnight at room temperature in CCF2 development media [19]. Cells were analyzed by flow cytometry using the NovoExpress software (version Windows 10).

### 2.9. Binding Assay

One million HuT 78 or primary CD4+ T cells were incubated in complete RPMI at 4 °C for 1 h and exposed to viral particles (100 ng p24 equivalent) for 30 min at 4 °C. After several washes with PBS, cells and bound viruses were centrifuged at 1200 rpm for 5 min. Pellets were lysed in 0.5% NP40/PBS, and viruses were quantified by p24 ELISA (Bio-techne, DY7360-05).

### 2.10. Single-Round Infection

Half a million HuT 78 or primary CD4+ T cells were infected with viral particles (500 ng p24 equivalent) for 5 h at 37 °C. After five washes in PBS, cells were incubated at 37 °C. Supernatants were collected 24 h post infection and quantified to p24 ELISA (Bio-techne, DY7360-05).

### 2.11. RRE Activity Assay

This assay was performed as described previously [16]. HEK-293T cells were co-transfected with pGag-RRE, pCMV-Rev, and pGFP. Cells were lysed 24 h after transfection, and Pr55Gag expression was quantified by Western blot. Results were normalized to β-actin and to GFP expressions.

### 2.12. Statistical Analysis

Statistical analyses were carried out with R Statistical Software (v4.5.0; R Core Team 2025, Vienna, Austria) and with Graph Pad Prism software version 10 in collaboration with the technical facility StatABio, BioCampus, UAR 3426, CNRS, Inserm, University of Montpellier, Montpellier, France. Differences among groups were tested by mixed-effect models of the *nlme* R package (v4.5.0; R Core Team 2025), to take into account for block effect of cell line batches and donors, where needed [20,21]. When samples size was large enough, we performed a normality test (Shapiro) to evaluate if a parametric test was applicable, and in that case, we performed the mixed-effect model. If the normality was not confirmed or samples were too small, we applied the paired non-parametric Wilcoxon signed-rank test in the case of paired samples with no block effect or a Wilcoxon for independent samples in the case of unpaired samples with no block effect. Otherwise, data were analyzed by mixed-effect models after being transformed into rank. If the original data and the rank transformed data produced comparable results, we considered that there was no violation of model assumptions and the results of the mixed-effect models were reported [22]. *p* values of < 0.05, 0.01, and 0.001 compared with the controls were considered significant and were noted as *, **, and ***, respectively. Error bars indicate standard error of the mean (SEM).

## 3. Results

### 3.1. Impact of ASP Expression on Cell–Cell Fusion

**Figure 1:**
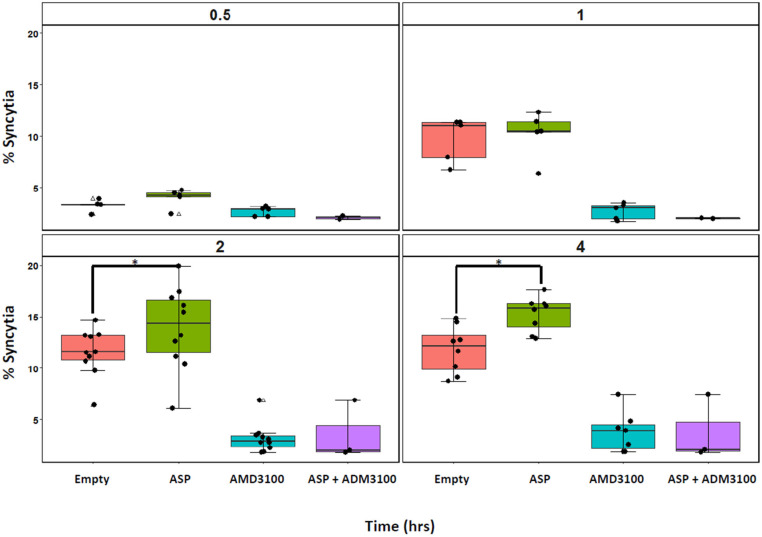
ASP enhances cell–cell fusion. HEK-Env-mCherry cells transfected with empty pCAGSS or pCAGSS-ASP were co-cultured with HuT 78-GFP in the presence or absence of AMD3100. Syncytia formation was quantified after 0.5, 1, 2, and 4 h. Double labeling was analyzed by flow cytometry. Triangles correspond to the outliers, *p* values of < 0.05, 0.01, and 0.001 compared with the controls were considered significant and were noted as *, **, and ***, respectively.

A previous study reported that, during viral replication, ASP localizes at the plasma membrane where it is detectable in close proximity of gp120 [12]. Thus, we sought to assess the hypothesis that plasma membrane-associated ASP plays a role in HIV-1 envelope-mediated fusion. We first used a transient co-culture system to monitor the formation of syncytia. We engineered a co-culture model combining, on the one hand, HEK-293T cells constitutively expressing the CXCR4-tropic envelope glycoproteins gp120/gp41 and the fluorescent protein, mCherry (HEK-Env-mCherry) and, on the other hand, HuT 78-GFP cells expressing CD4, CXCR4, and GFP (Figure S1). The two cell lines were co-cultured at a 1:1 ratio to validate our system via observation and quantification of syncytia by fluorescence microscopy and flow cytometry (Figure S1). Following this validation, we conducted several controls. First, we assessed the formation of syncytia in the presence of AMD3100, which antagonizes CXCR4 and prevents interaction with gp120 [23]. We did not observe any cell fusion in the presence of AMD3100, regardless of whether the HEK-Env-mCherry cells expressed ASP. Next, we evaluated whether the expression of ASP alone in the absence of Env expression could induce syncytia formation. For this purpose, parental HEK 293T cells were transfected with either an empty vector or a vector expressing ASP. Cell fusion and syncytia formation were not observed by light microscopy after 24 h of co-culture with HuT 78 cells (Figure S2). Subsequently, we investigated the subcellular localization of ASP in HEK 293T-Env cells transfected with the optimized pCAGGS-ASP plasmid (Figure S3). Finally, HEK-Env-mCherry cells were transfected with either an empty vector or a vector expressing ASP, and then they were co-cultured with HuT 78-GFP cells. Syncytia formation was assessed after 30 min, 1 h, 2 h, and 4 h of co-culture. At 2 and 4 h post co-culture, we observed a significant increase in the percentage of syncytia in co-cultures containing HEK293T-Env-mCherry expressing ASP compared with the control co-cultures (*p* = 0.0273 at 2 h and 4 h post co-culture) (Figure 1 and Figure S4). Moreover, our analyses showed an overall trend toward increased syncytium size at 4 h post co-culture in the presence of ASP compared with the control cultures, suggesting a higher number of cell–cell fusion events (Figure S1).

### 3.2. Impact of ASP on the HIV-1 Replication Cycle When Expressed to the Surface of Viral Particles

**Figure 2:**
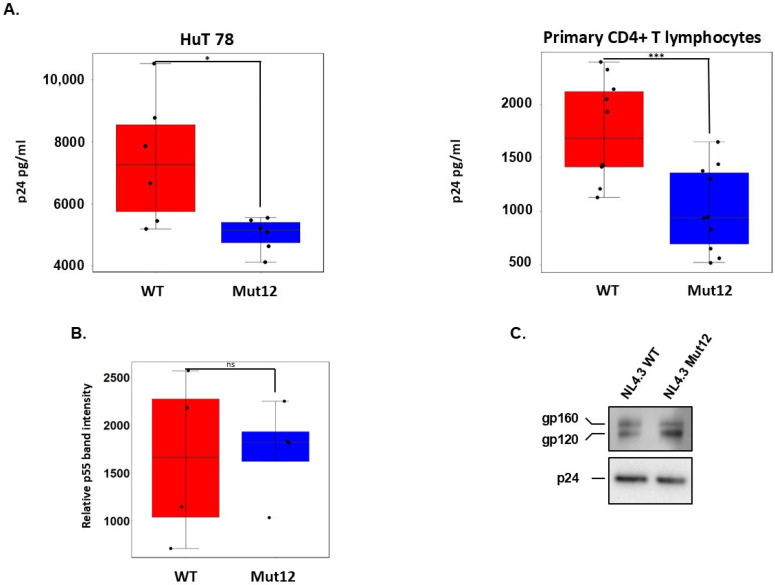
Viral production is increased by HIV-1 antisense protein ASP. (**A**) HuT 78 cells or primary CD4+ T cells were infected with 500 ng (p24 equivalents) of NL4-3 WT or NL4-3 Mut12. Supernatants were collected 24 h post-infection, and extracellular p24 levels were quantified by enzyme-linked immunosorbent assay (ELISA). For the primary CD4+ T cells, analyses were carried out on cells obtained from 5 different donors. (**B**) RRE activity was assessed by quantifying the Pr55Gag expression by Western Blot in HEK 293T transfected with RRE-WT or RRE-Mut12 expression vectors as well as a GFP expression vector. (**C**) Expression levels of envelope proteins on viral particles were monitored by Western blot. Results shown are representative of 6–8 different viral stocks. *p* values of < 0.05, 0.01, and 0.001 compared with the controls were considered significant and were noted as *, **, and ***, respectively; ns = non statistically significant.

Previous studies showed that ASP is expressed on the envelope of infectious HIV-1 virions [12]. Therefore, we sought to explore the functional contribution of ASP during the course of the viral life cycle. We generated an ASP-knockout (ASP-ko) HIV-1 NL4-3 molecular clone by introducing a synonymous nucleotide substitution at codon 559 of *env* that created a stop at codon 12 of the ASP ORF (Mut12; TGC > TGA) [24] but did not change the amino acid sequence of the Env protein. To confirm that such mutation abolished ASP expression, we introduced the TGC > TGA substitution in a vector expressing an ASP-GFP fusion protein. The vectors carrying the wildtype ASP ORF (WT) and the ASP Mut12 ORF (ASP-ko) were then transfected into HEK293T cells, and whole cell lysates were probed with anti-ASP and with anti-actin antibodies. The results shown in Figure S5 confirm that the TGC > TGA substitution (Mut12) blocks ASP expression. Next, the WT and ASP Mut12 NL4-3 viral strains were produced by transfecting the respective molecular clones into HEK293T cells, and then they were used to assess the impact of ASP expression in single-round infection assays. HuT 78 cells and primary CD4+ T lymphocytes from healthy donors were infected with equal p24 equivalents of the WT or ASP Mut12 viral stocks, and the extracellular level of p24 in culture supernatants were then quantified 24 h after infection. As shown in Figure 2A, significantly lower p24 levels were observed in culture supernatants of cells infected with ASP-knockout Mut12 viruses compared with WT (linear mixed-effect model; *p* = 0.0247 for HuT 78; *p* < 0.0001 for primary CD4+ T cells), indicating that the ASP expression might play a role in the HIV-1 replication cycle. Given that the *asp* gene overlaps the HIV-1 Rev Response Element (RRE), we assessed whether the nucleotide substitution that creates the stop codon at position 12 of *asp* had an impact on the activity of RRE. Thus, HEK293T cells were co-transfected with three plasmids: (1) a Rev-dependent Gag-Pol expression vector containing either the wildtype RRE sequence (pGag-Pol-RRE) or a RRE sequence carrying the Mut12 nucleotide substitution (pGag-Pol-Mut12-RRE); (2) a vector expressing the Rev protein (pRev), and (3) a vector expressing green fluorescent protein (pGFP). After transfection, cell-associated Pr55Gag levels were analyzed by Western blot in whole cell lysates prepared from the two cultures. The results shown in Figure 2B indicate that the Mut12 nucleotide substitution did not significantly affect RRE activity compared with the wildtype sequence (*p* = 0.848). Moreover, as ASP was present on the envelope of viral particles [12], we explored the possibility that the lack of ASP might affect incorporation and cleavage of Env glycoprotein. Western blot analyses of virion-associated Env glycoprotein did not show any difference in the incorporation, proteolytic processing, or stability of Env in WT compared with ASP Mut12 viral particles (Figure 2C).

**Figure 3:**
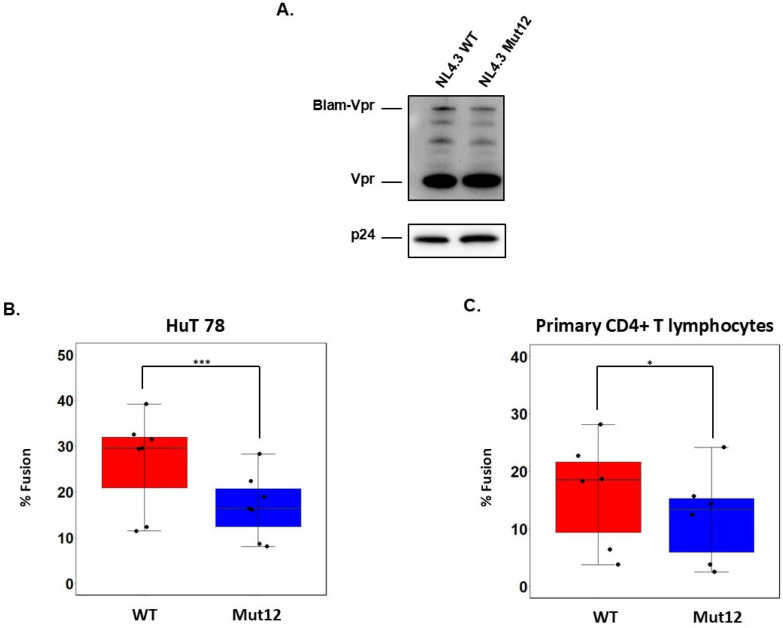
ASP promotes HIV-1 fusion. (**A**) Incorporation of Blam-Vpr into NL4.3 WT or NL4.3 Mut12 virions was determined by Western Blot. The level of p24 was used to normalize the amounts of viral particles for infection. HuT 78 cells (**B**) or primary CD4+ T cells (**C**) were mock-infected or infected with either NL4.3 WT or NL4.3 Mut12 containing Blam-Vpr for 3 h at 37 °C. Viral entry was quantified by flow cytometry using violet laser to excite CCF2. *p* values of < 0.05, 0.01, and 0.001 compared with the controls were considered significant and were noted as *, **, and ***, respectively.

To gain deeper insight into how viruses carrying ASP contribute to the HIV-1 replication cycle, we examined the impact of ASP on viral entry using the BlaM-Vpr assay, a standard method allowing quantification of viral fusion [18,19]. We co-transfected HEK293T cells with the WT or ASP Mut12 molecular clones along with a vector expressing the Vpr-β-lactamase fusion protein (Vpr-BlaM). After purification and concentration of the two viral stocks, we first verified equal incorporation of Vpr-BlaM (Figure 3A). Next, 1 μg (p24 equivalents) of the two viral stocks were added to HuT 78 labeled with CCF2-AM. After incubation for 3 h at 37 °C, we measured the resulting fluorescence signal by flow cytometry. We found that infection with ASP Mut12 viral stock resulted in a significant drop of cleaved CCF2 cells compared with WT (linear mixed-effect model; *p* = 0.0002; Figure 3B). Similar results were obtained using primary CD4+ T lymphocytes from healthy donors (paired Student test; *p* = 0.0102; Figure 3C). The gating strategy and representative flow cytometry plots are shown in Figure S6. These findings suggest that expression of ASP on the viral envelope enhances HIV-1 entry.

**Figure 4:**
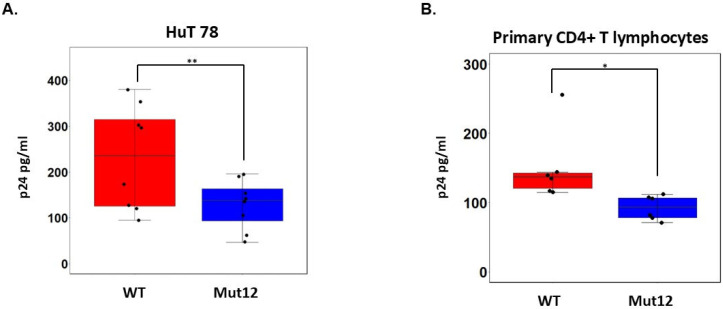
ASP facilitates HIV-1 attachment to target cells. (**A**) Attachment of wildtype or ASP ko viruses was carried with 1´106 HuT 78 cells exposed to 100 ng (p24 equivalents) of viruses for 30 min at 4 °C. After removal of unbound viruses, bound viruses were lysed and quantified by p24 ELISA. (**B**) Attachment of wildtype or ASP ko viruses was carried with 1´106 primary CD4+ T cells exposed to 100 ng (p24 equivalents) of viruses for 30 min at 4 °C. After removal of unbound viruses, bound viruses were lysed and quantified by p24 ELISA. *p* values of < 0.05 and 0.01 compared with the controls were considered significant and were noted as * and **, respectively.

Given these results, we performed binding assays to determine whether ASP expression on the viral envelope affects attachment of the virus to HuT 78 or primary CD4+ lymphocytes. The results shown in Figure 4 show that ASP Mut12 viruses exhibit a significantly lower binding capacity compared with WT viruses (HuT 78 cells: Wilcoxon signed-rank test; *p* = 0.007813; primary CD4+ T cells: linear mixed-effect model; *p* = 0.0328).

Altogether, our studies suggest that the presence of ASP on the surface of the viral particles facilitates viral attachment to target cells and consequently enhances viral entry.

## 4. Discussion

The *asp* ORF originated recently, when group M HIV-1 strains diverged from their most immediate progenitor, SIVcpz_Ptt [2,3,5]. Creation of the *asp* gene occurred via gradual loss of premature stop codons in the genomic region containing the ORF in viral strains that were progressively closer to HIV-1 group M [2,3,5]. This suggests the possibility that the creation and conservation of a full-length *asp* ORF may be beneficial to HIV-1 by facilitating viral spread or pathogenesis.

To date, no function has been associated with ASP. Here, we present, for the first time, evidence suggesting that ASP may play a functional role in the HIV-1 life cycle. Through the use of viral strains that do or do not express ASP, we show that ASP facilitates viral attachment and entry in CD4+ T lymphocytes.

Today, only two antisense proteins have been described in retroviruses: HBZ of the human T-cell lymphotropic viruses (HTLV-1, 2, 3 and 4) and ASP of HIV-1. In the case of HTLV-1, HBZ has been shown to play a critical role in the virus life cycle and in the pathogenic process. HBZ inhibits HTLV-1 sense transcription by recruiting essential transcription factors and also affects several cellular processes including host gene expression, innate immune signaling, apoptosis, autophagy, and DNA repair [5,25,26,27]. Therefore, identifying a functional role for ASP is particularly important to gain new insights into the natural history of HIV-1 infection. The great majority of novel viral proteins are “accessory” or “auxiliary” (i.e., neither structural nor enzymatic) and originated via overprinting a structural or enzymatic gene. Notably, the terms “accessory” or “auxiliary” do not indicate proteins that have no impact on viral replication. Rather, such proteins play an important role in viral pathogenicity or spread [25]. Thus, de novo proteins like ASP appear to be significant factors in viral evolution. It is highly likely that, like all HIV-1 proteins classified as accessory or auxiliary, ASP performs multiple functions throughout the HIV-1 replication cycle, making the elucidation of ASP functions particularly challenging due to its potential cumulative effects.

At this stage, the exact mechanism by which ASP promotes the viral entry remains to be elucidated. Future experiments will provide important mechanistic insights and reveal the processes that ASP exploits to enhance viral entry. Understanding the role of ASP in HIV-1 infection may suggest potential new targets for future antiviral therapeutics.

## Data Availability

Data availability requests can be fulfilled by contacting the corresponding author.

## Supplementary Materials

The following supporting information can be downloaded at https://www.mdpi.com/article/10.3390/v17101332/s1, Figure S1: syncytia formation, Figure S2: analysis of ASP expression over the time, Figure S3: analysis of ASP WT and ASP Mut12 expression, Figure S4: Analysis of ASP expression over time, Figure S5: Analysis of ASP WT and ASP Mut12 expression, Figure S6: Gating strategy to measure HIV-1 fusion to HUT78 cells.
